# Behavior Change Support Systems for Self-Treating Procrastination: Systematic Search in App Stores and Analysis of Motivational Design Archetypes

**DOI:** 10.2196/65214

**Published:** 2025-02-20

**Authors:** Jeanine Kirchner-Krath, Manuel Schmidt-Kraepelin, Katharina Schmähl, Christoph Schütz, Benedikt Morschheuser, Ali Sunyaev

**Affiliations:** 1 School of Business, Economics and Society Friedrich-Alexander-Universität Erlangen-Nürnberg Nuremberg Germany; 2 Department of Economics and Management Karlsruhe Institute of Technology Karlsruhe Germany; 3 Gamification Research Group, Faculty of Information Systems and Applied Computer Sciences University of Bamberg Bamberg Germany; 4 School of Computation, Information, and Technology Technical University of Munich Campus Heilbronn Heilbronn Germany

**Keywords:** procrastination, mobile apps, cluster analysis, motivational design, persuasive technology, behavior change support systems, behavior change techniques, mobile health, mHealth, mobile phone, artificial intelligence, AI

## Abstract

**Background:**

The phenomenon of procrastination refers to an individual’s conscious decision to postpone the completion of tasks despite being aware of its adverse consequences in the future. Extant research in this field shows that procrastination is associated with increased levels of anxiety and stress and the likelihood of developing depression and calls for the development of suitable interventions that support individuals in making lasting positive changes to their procrastination behaviors. In parallel, practice has produced a plethora of behavior change support systems (BCSSs) that aim to provide a low-threshold, accessible alternative to in-person therapeutic approaches. Most of these BCSSs can be considered motivational BCSSs that combine functional, utilitarian components with hedonic and eudaimonic design elements to empower self-treatment. Although early studies have suggested the potential benefits of such BCSSs, research on understanding their specific design characteristics and support of individuals in self-treating procrastination is still in its infancy.

**Objective:**

In response to this gap between practice and research, we aimed to analyze and systemize the multitude of practical design efforts in motivational BCSSs for the self-treatment of procrastination and identify the main design archetypes that have emerged.

**Methods:**

We conducted a 3-step research approach. First, we identified 127 behavior change support apps for procrastination through a systematic screening process in the German and US Apple App Store and Google Play Store. Second, we systematically coded the identified apps in terms of the behavior change techniques targeted by their functional design and hedonic or eudaimonic design elements. Third, we conducted a 2-step cluster analysis to identify archetypes of motivational design in behavior change support apps to combat procrastination.

**Results:**

A variety of motivational designs have been developed and implemented in practice, and our analysis identified five main archetypes: (1) structured progress monitor, (2) self-improvement guide, (3) productivity adventure, (4) emotional wellness coach, and (5) social focus companion. The identified archetypes target different psychological determinants of procrastination and successfully use a variety of hedonic and eudaimonic design elements that extend beyond the current state of research.

**Conclusions:**

The results of our study provide a foundation for future research endeavors that aim to examine the comparative effects of motivational design archetypes and develop more effective interventions tailored to individual needs. For practitioners, the findings reveal the contemporary design space of motivational BCSSs to support the self-treatment of procrastination and may serve as blueprints that can guide the design of future systems. For individuals seeking support and health professionals treating procrastination, our study systemizes the landscape of apps, thereby facilitating the selection of one that best aligns with the patient’s individual needs.

## Introduction

### Motivation

Procrastination, an irrational and malicious behavioral pattern defined as the intentional delay of important or necessary tasks despite knowing the negative consequences [1], affects nearly one-fifth of adults worldwide [2,3]. Despite the phenomenon often being described with a bit of humor, as evidenced by a popular Technology, Entertainment, Design Talk [4], it poses a serious threat to mental and physical health. Studies have demonstrated that procrastination can cause stress, depression, and anxiety [5-7], as well as raise the risk of cardiovascular diseases and hypertension [8]. Overall, those affected by procrastination experience a concerning risk of a decline in life quality [9].

Correspondingly, medical research has engaged in numerous treatment directions to help and support those affected [10,11]. Acknowledging that procrastination primarily results from a failure of self-regulation [2], which is influenced by both situational (eg, task characteristics, expected outcome, and delay) and motivational (eg, self-efficacy, self-belief, and impulsiveness) determinants [12], cognitive and behavioral interventions as well as their combination in cognitive behavioral therapy promise to be most successful [10,13-15]. However, traditional treatment of procrastination still suffers from low attendance and adherence rates compared to the amount of people affected [16]. In this regard, digitally supported treatment of procrastination using behavior change support systems (BCSSs) has gained attention as a low-threshold, accessible alternative to in-person therapeutic approaches [11,14,17].

BCSSs are systems “with psychological and behavioral outcomes designed to form, alter or reinforce attitudes, behaviors or an act of complying without using coercion or deception” [18]. They have been demonstrated to help restructure malicious behaviors that pose health threats [19], such as obesity [20-22], lack of physical activity [23,24], unhealthy nutrition [25-27], or smoking [28-30]. Through various behavior change techniques (BCTs) that target situational and motivational behavior determinants, such as goal setting and self-monitoring, context restructuring, reframing of beliefs, or the illustration of behavioral consequences [31], BCSSs can support a stepwise transition from health-threatening behaviors to novel behavior patterns and habits [32]. A recent review of primary studies in the field of BCSSs for procrastination [17] demonstrates that such BCSSs can evidently help raise awareness of distractions [33-35], reduce procrastination behaviors [34,36,37], and increase self-efficacy [37-39].

While these studies are still often tied to augmenting traditional cognitive behavioral therapy with digital applications under medical guidance, it is only in the last few years that BCSSs with motivational design approaches for the successful self-treatment of procrastination have gained traction [17]. This development can be attributed to the emerging research trend of motivational information systems (ISs) [40,41], which combine functional, utilitarian components (such as support in planning and goal setting) with hedonic (referring to a sense of pleasure [42]) and eudaimonic (referring to a sense of meaningfulness [42]) design elements. The latter aim to evoke positive experiences in using the system and performing the target behavior, such as joy, excitement, or curiosity, and satisfy motivational needs [40,41]. Initial studies show that motivational BCSSs for procrastination that combine educational content about procrastination stimuli with quiz and achievement mechanics can significantly reduce procrastination in unguided self-treatment [43]. Moreover, gameful storytelling in task planning and work on tasks can enhance feelings of self-control [44].

Research on motivational BCSSs for low-threshold and accessible self-treatment of procrastination is still nascent, but practical efforts have produced a plethora of (primarily mobile) apps that use motivational design approaches to support people in handling procrastination behaviors [44]. These include gamified task management platforms that integrate narrative design [45]; artificial companions that provide approval and help with planning and self-reflection [46]; and digital tools that use focus timers, unlockable collectibles, and team focus sessions [47], with several apps exceeding a million downloads. In this regard, there is great potential for research on motivational BCSSs for procrastination to build on and learn from these practical efforts [17] and investigate how different motivational design approaches affect the procrastination process as a basis to develop targeted recommendations for individuals seeking support in dealing with procrastination.

In this context, previous research has demonstrated that effectively designing health BCSSs requires not only experimenting with different BCTs that, in untargeted combinations, can cause counteracting mechanisms and result in highly ineffective solutions [48]. Rather, to provide an informed foundation for both researchers and designers on how to successfully approach the problem of procrastination with motivational BCSSs, it is crucial to identify and characterize commensurate combinations of BCTs and take account of their underlying theoretical mechanisms [48]. To this end, identifying the main *archetypes* of design that have emerged to target situational and motivational determinants of procrastination in practical applications is particularly valuable. Design archetypes represent a practice-oriented perspective on design knowledge that *abstracts* from single applications, which might sometimes combine BCTs randomly, to identify industry best practices. Thus, design archetypes can be regarded as complementary to conceptual, theory-driven design knowledge (eg, design principles and frameworks) in guiding developers when designing and implementing motivational BCSSs by offering prescriptions for effective design [49]. For researchers, knowledge about design archetypes further serves as a feedback mechanism that shows whether and, if so, how research insights have been transferred to real-world systems and where gaps between research and practice remain [50].

### Objectives

This study aimed to provide this foundation for both future research in investigating motivational BCSSs to combat procrastination and further practical efforts to design and select motivational BCSSs for self-treating procrastination by answering the following research question: *what are prevalent archetypes of motivational design in behavior change support apps for procrastination?*

To answer this research question, in line with previous studies that have systematized design in (mobile) health apps [50-52], we adopted a systematic screening and coding approach that built on the recognized Behavior Change Technique Taxonomy (BCTT) by Michie et al [53] and the intrinsic motivations for system use classification by Lowry et al [54] to form the basis for a 2-step cluster analysis according to Punj and Stewart [55]. Thereby, we identified clusters of motivational design approaches in prevailing motivational BCSSs for procrastination and then discussed the archetypes of motivational design characteristics for each identified cluster.

Consequently, our study systematically classified motivational design approaches in BCSSs to combat procrastination, providing 3 main contributions to research and practice. First, it constitutes the basis for future research efforts to investigate the comparative effects of motivational design archetypes on various situational and motivational determinants of procrastination, possibly paving the way for more targeted and individualized self-treatment recommendations through motivational BCSSs depending on the individual problem profile [1,56,57]. Second, it represents blueprints of design opportunities for developers of BCSSs to support the self-treatment of procrastination using motivational design approaches that increase system use and counteract dropout rates, a problem known from health-related digital applications [58]. Finally, it systemizes the plethora of available apps for both health professionals and individuals seeking support in handling procrastination as a basis to decide on a design archetype that best suits the patient’s or individual’s needs and preferences, thereby potentially increasing treatment adherence and counteracting the severe mental and physical health consequences of ongoing procrastination.

## Methods

### Overview

**Figure 1:**
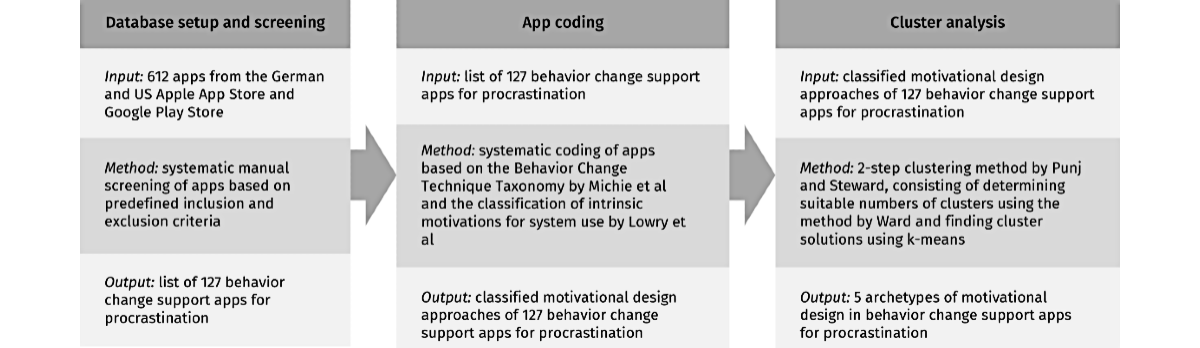
Overview of the 3 research steps, applied research methods, and outputs of the methods.

To answer our research question, we followed a systematic screening and coding approach of existing mobile apps, complemented by a 2-step cluster analysis [55] informed by previous work that has conducted archetype analyses of health-related apps and platforms [50-52]. Accordingly, our research method consisted of 3 main steps: database setup and screening, app coding, and cluster analysis (Figure 1 [50-55]).

### Database Setup and Screening

**Figure 2:**
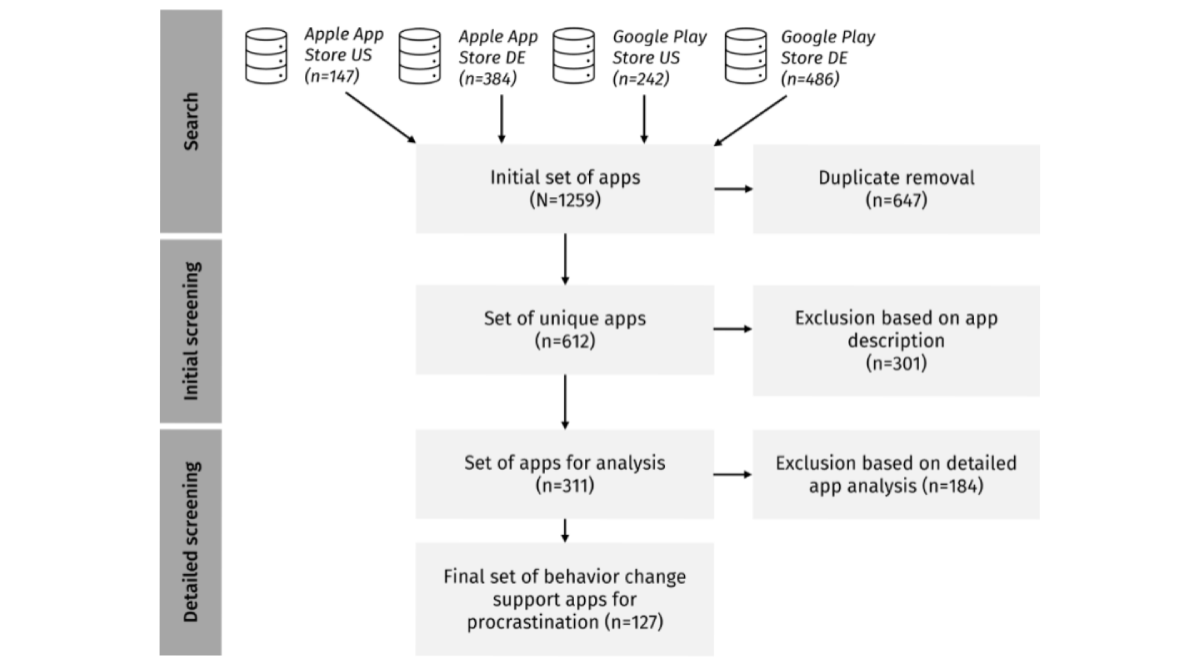
Overview of the app screening and selection process. DE: Germany; US: United States.

To set up the database of pertinent behavior change support apps to combat procrastination, we followed a systematic search strategy in the German and US Apple App Store and Google Play Store. The reason why we opted for these 2 platforms is that they represent the 2 largest app stores for iOS- and Android-based mobile phones, with the Google Play Store recording >110 billion app downloads and the Apple App Store reporting approximately 35 billion app downloads in 2023 [59]. Moreover, the United States is the most significant Western mobile app market in terms of downloads and consumer spending on mobile devices [60], and Germany represents the largest mobile app market in the European Union [61]. We searched for apps related to the terms “procrastination” or “Prokrastination” (the German equivalent) in March 2024, leading to an initial set of 1259 apps (Figure 2).

After removing duplicates, of the 1259 apps, 612 (48.61%) unique apps remained for the initial screening. Thus, we screened the descriptions of these 612 apps in the Apple App Store and Google Play Store against our predefined inclusion and exclusion criteria, listed in Textbox 1.

**Inclusion and exclusion criteria for the screening and selection process.**

**Inclusion criteria**

- Topicality: maintained apps with regular updates
- Access: free apps
- Language: English or German
- Content focus: explicit focus on the problem of procrastination at work or in daily life
- App design: motivational behavior change support system with both functional and hedonic or eudaimonic design aspects according to the classification of intrinsic motivations for system use by Lowry et al [54]

**Exclusion criteria**

- Topicality: outdated apps flagged as not maintained anymore
- Access: paid apps and paywalls after installation
- Language: other languages
- Content focus: lack of focus on procrastination (eg, household organizers; microlearning apps; time-tracking apps for client projects; team planners; general meditation apps; general habit trackers; mood trackers; and treatment apps for other mental health issues such as attention-deficit/hyperactivity disorder, depression, or anxiety)
- App design: purely functional, utilitarian app design without hedonic design aspects

Specifically, concerning their topicality, we excluded apps flagged as not maintained by the app stores. We also excluded paid apps that were not accessible without upfront payment as we wanted to focus on apps that are readily accessible to a broad audience of people affected by procrastination without posing a payment hurdle that might inhibit their benefit compared to traditional treatment approaches. Moreover, we excluded apps that were in languages other than English or German (eg, Chinese or Russian). In terms of their content and focus, we explicitly searched for apps that targeted procrastination of individuals at work or in daily life by providing specific utilitarian functionalities or content to approach procrastination (ie, the postponement of important or necessary tasks) on a cognitive (eg, specific learning or reflection content or guidance related to procrastination) or behavioral (eg, features to engage in goal and task planning or organization or focused work) level. Conversely, we excluded apps that were not focused on this problem, such as microlearning apps and general meditation apps (with content entirely unrelated to procrastination); household organizers; time-tracking apps for client projects or team planners (focused on organizing multiple people instead of supporting individuals); mood trackers and general habit trackers (without any content or functionalities related to specific tasks); or treatment apps for other mental health problems such as attention-deficit/hyperactivity disorder, depression, or anxiety (that were not suitable for individuals without pathological mental health issues). Moreover, as we focused on motivational BCSSs that are characterized by functional, utilitarian, and hedonic or eudaimonic design aspects [40,41], we excluded apps without any visible hedonic or eudaimonic design approaches, such as purely functional to-do lists, calendars, timers, or app blockers.

As a result of the initial screening process, of the 612 apps, 311 (50.8%) remained for the subsequent detailed analysis. In this step, we downloaded all the apps on an iPhone 15 Pro (for iOS apps) and a Google Pixel 4a (for Android apps) and analyzed their functionalities in detail against the predefined inclusion and exclusion criteria. During this step, we excluded an additional 59.2% (184/311) of the apps because they did not meet the inclusion criteria. Specifically, of the 184 apps, 34 (18.5%) were excluded because they had a paywall for use after installation (n=32, 94%) or could be accessed only via invitation (n=2, 6%); 16 (8.7%) were excluded because they were not usable (ie, they had been either removed from the app stores between screening in March 2024 and analysis in May 2024, n=6, 38%, or were buggy so that the main functionalities could not be used, n=10, 62%); 3 (1.6%) were excluded because they were only available in Turkish, French, or Chinese; 70 (38%) were excluded because they were not focused on the problem of procrastination; and 51 (27.7%) were excluded because they did not exhibit any hedonic design approaches. To decide whether an app entailed hedonic or eudaimonic design approaches, we juxtaposed them with our codebook based on the classification of intrinsic motivations for system use by Lowry et al [54] (Table 1) and excluded those apps that did not provide hedonic or eudaimonic features to evoke any of these defined motivations.

**Table 1:** Codebook of hedonic and eudaimonic design aspects based on the classification by Lowry et al [54].

Design aspect (based on the classification by Lowry et al [54])	Definition based on the Cambridge Dictionary	Exemplary design features (added after intercoder agreement check)
Fun and enjoyment	Experience of playfulness or pleasure	Mini games, role-play, digital companions, juicy animations and sounds, or shuffling or chance
Escapism	Experience of escaping pressures and avoiding an unpleasant or boring life	Fantasy, reframing, or virtual reality
Challenge	Experience of being faced with something that requires mental or physical effort and tests one’s ability	Quests or daily challenges, leaderboards, or levels
Immersion	Experience of becoming completely involved in an environment or action	Story or narrative, nature sounds, or meditations
Curiosity	Experience of being eager to know or learn about something or encountering something interesting, rare, or unusual	Counterintuitive behavior or surprising events
Discovery	Experience of finding information, places, or objects that one did not know about before	Easter eggs, skill paths, unlockable content, or new content
Self-expression	Experience of expressing one’s personality, emotions, or ideas	Personal profile, personal journal, or customization of an avatar
Knowledge expansion and self-development	Experience of learning, growing, changing, or advancing through one’s own efforts	Skill trees, quizzes, comparison to past behavior, or self-reflection
Freedom and autonomy	Experience of being allowed to do, say, and think what one wants and make one’s own decisions without being controlled or limited by anyone else	Choices, customization and configuration, or multiple paths and options
Companionship	Experience of enjoyment in spending time with other people or being bound with others that share similarities	Collaboration partner, social feed, digital coffee, or community
Approval	Experience of receiving positive opinions, being accepted, or being positively reinforced for one’s actions	Motivational messages, positive feedback, likes and comments, or badges
Meaning	Experience of importance or value in an action or outcome	Narrative, coupling actions with benefits for others, altruism, or gifting

To ensure the reliability of our exclusion process, 3 authors took part in the detailed screening. One of them was a postdoctoral researcher in motivational ISs with 5 years of research experience in the field of BCSSs and motivational design, who set up the inclusion and exclusion criteria, defined the codebook, and led the screening and selection process. The 2 others were junior researchers with backgrounds in organizational psychology and business pedagogy. To ensure reliability, an intercoder agreement check was carried out after 40.2% (125/311) of the apps were analyzed. A total of 20% (25/125) of the analyzed sample was randomly selected and additionally analyzed by one of the other authors (specifically, the apps analyzed by the junior researchers were subjected to intercoder checking by the postdoctoral researcher, and the apps analyzed by the postdoctoral researcher were equally divided and checked by the 2 junior researchers). The intercoder agreement rate for inclusion and exclusion was 96% (κ=0.92), indicating nearly perfect agreement [62]. Any conflicts were resolved in a joint discussion, and the 3 authors progressed with the remaining apps. As a result, 127 apps remained as the final set of behavior change support apps for procrastination for the following research step. The complete list of included and excluded apps can be found in Multimedia Appendix 1.

### App Coding

For our app coding as a basis to identify motivational design archetypes, we opted for 2 existing classifications to code the utilitarian, functional design aspects and the hedonic or eudaimonic design aspects of the apps, which are both crucial for motivational ISs [40,41].

Regarding the functional design, we used the BCTT by Michie et al [53]. Michie et al [53] developed a taxonomy of 93 different BCTs organized in 16 hierarchical clusters*: goals and planning*, *feedback and monitoring*, *social support*, *shaping knowledge*, *natural consequences*, *comparison of behavior*, *associations*, *repetition and substitution*, *comparison of outcomes*, *rewards and threats*, *regulation*, *antecedents*, *identity*, *scheduled consequences*, *self-belief*, and *covert learning* [63]. The BCTT has proven its reliability and validity [31] and has already been widely used in studies to classify health applications and digital health interventions [19,64-68]. While alternative taxonomies for classifying design in BCSSs exist, such as the persuasive system design (PSD) model [69], which has also been extensively applied in research on health BCSSs [70,71], we decided to build on the BCTT for 2 reasons. First, unlike the PSD model, it incorporates cognitive techniques such as identity and self-belief. Given that previous IS research in the field of motivational BCSSs for self-treating procrastination has largely disregarded the use of cognitive techniques [17], it is all the more important to analyze whether such approaches have been incorporated into the practical design of motivational BCSSs. Second, PSD incorporates principles that are on the cusp of being considered utilitarian, such as liking (which refers to the aesthetic experience and, thus, is related to the hedonic experience of enjoyment [41]) or competition and praise (which can be considered hedonic elements aiming to evoke experiences of challenge and approval [54]). However, it does not encompass the full range of hedonic and eudaimonic aspects that could be included in apps, such as immersion, curiosity, or discovery [54]. Accordingly, we chose to use the BCTT as a taxonomy to classify the functional design of the analyzed apps complemented by a second classification explicitly targeting the hedonic and eudaimonic aspects of the apps. Consequently, for our analysis, we coded the apps based on the 16 overarching clusters of the BCTT; for example, an app was coded as meeting the BCT cluster *self-belief* if it provided functionalities for (1) verbal persuasion about capability, (2) mental rehearsal of successful performance, (3) focus on past success, or (4) self-talk, whereby the detailed definitions and examples for each BCT provided by the BCTT [63] served as our codebook.

In terms of the hedonic and eudaimonic design aspects of the apps [41], we opted for the classification of intrinsic motivations for system use provided by Lowry et al [54]. In their study, Lowry et al [54] list 11 intrinsic motivations for hedonic IS use based on a review of the IS literature: *fun and enjoyment*, *escaping pressures*, *challenge*, *immersion*, *curiosity*, *discovery*, *self-expression*, *obtaining knowledge*, *experiencing autonomy/freedom*, *peer companionship*, and *approval* [54]. According to their hedonic-motivation system adoption model, which has been extensively used to evaluate motivational BCSSs in the health domain [72-75], these hedonic motivations are pivotal to the adoption and continued use of systems that target hedonic experiences [54]. We defined each of these intrinsic motivations based on the Cambridge Dictionary and added *meaning* (ie, a sense of experiencing importance or value in an action [76]) as the 12th dimension to reflect the eudaimonic aspect of motivational ISs [40]. During the coding process, an app was coded to meet a specific dimension if it provided functionalities to evoke the described hedonic or eudaimonic experience (eg, of fun or challenge; Table 1).

In total, 3 authors performed the coding independently. Each app was downloaded, tested, and used to experience all features and functionalities. In this regard, we used each app intensively upon installation (ie, we clicked on all menu items, buttons, and learning content and used all available functionalities on the app to experience whether completing tasks or using features on the app resulted in system feedback or progress), and if the app included progression mechanics to unlock new features or notification and reminder mechanics, we also used the apps for several days to code all their features that might become visible over time. To ensure reliability in the coding process, we performed an intercoder agreement check after coding 30% of the apps, again randomly selecting 25 apps for intercoder checks and dividing the intercoder checks in a similar way to that for the screening check (ie, the apps coded by the junior researchers were subjected to intercoder checking by the postdoctoral researcher, and the apps coded by the postdoctoral researcher were equally divided and checked by the 2 junior researchers). The intercoder agreement rate ranged between 50% and 100% for the BCTs (with a mean agreement rate of 84%, SD 14%) and between 67% and 92% for the hedonic and eudaimonic design aspects (with a mean agreement rate of 82%, SD 6%), indicating good agreement [62]. As a result of the joint discussion, we streamlined our understanding of the BCTs (eg, defining that the social support technique only refers to support from other human beings and not from artificial agents on the app) and enlarged our codebook of hedonic and eudaimonic design aspects based on exemplary design features (Table 1) before progressing with the coding process. The final list of the 127 coded apps can be found in Multimedia Appendix 1.

### Cluster Analysis

We used cluster analysis to derive meaningful archetypes of app designs in the third step of our methodology. To do so, we followed the 2-step clustering approach proposed by Punj and Stewart [55] for 2 main reasons. First, this approach combines hierarchical clustering with iterative partitioning algorithms, thereby attempting to overcome the respective weaknesses of both approaches (eg, many partitioning algorithms, such as k-means, perform better than hierarchical algorithms but require an a priori definition of the number of clusters). Second, this methodology has been successfully applied to similar clustering tasks and has been shown to be useful for identifying design archetypes in various contexts [50,51,77].

In line with the approach by Punj and Stewart [55], our clustering analysis included 2 steps. In the first step, we used the method by Ward as a hierarchical clustering method to determine preliminary solutions and identify potential candidate numbers of clusters. Our coding included only binary data (ie, a design aspect, meaning each of the 16 BCT clusters of the BCTT [53] and each of the 12 hedonic or eudaimonic design aspects based on the classification by Lowry et al [54], is either present or absent). Thus, we chose the Euclidean squared distance as the similarity measure [50]. The dendrogram resulting from applying the method by Ward to our dataset indicated that 2 to 5, 7, 9, 11, and 12 were suitable candidate numbers of clusters. By examining the scree plot using the elbow rule [78], we narrowed down our selection to the 2-, 4-, 5-, 7-, 9-, or 12-cluster solution. On the basis of these candidate numbers, we conducted k-means clustering as an iterative partitioning algorithm. The 7-, 9-, and 12-cluster solutions produced by k-means all contained clusters with only 4 or even fewer apps, making reasonable interpretation of these solutions difficult. To decide on one of the remaining cluster solutions, 2 researchers manually compared their explanatory power by investigating differences and similarities in the BCTs and hedonic and eudaimonic design aspects included in our coding. First, we decided against the 2-cluster solution because the clusters were too heterogeneous in design aspects, making it impossible for us to derive meaningful interpretations. This left us with the 4- and 5-cluster solutions as candidates. After carefully analyzing both cluster solutions, we concluded that they were similar in many aspects but that one of the clusters in the 4-cluster solution was further divided into 2 distinct and informative clusters within the 5-cluster solution. Thus, we decided that the 5-cluster solution had the highest explanatory power and was the most suitable for deriving meaningful archetypes within this study. The cluster analysis results and the cluster solutions are included in Multimedia Appendix 2.

## Results

### Overview

The final apps (N=127) that we included were fairly homogeneous regarding their categories, with most (n=122, 96.1%) categorized as productivity apps. In addition, 1.6% (n=2) were listed as lifestyle apps, 1.6% (n=2) were labeled as health and fitness apps, and 0.8% (n=1) were subsumed under the category of education, suggesting that, from a developer’s perspective, apps that support behavior change to combat procrastination are seen as primarily organizational tools rather than mental health support apps. Overall, the analyzed apps were well rated (mean 4.39, SD 0.48 stars), with 1.6% (n=2) of the apps with a rating <3 stars, 11.8% (n=15) with a rating between 3 and 4 stars, 23.6% (n=30) with a rating between 4 and 4.4 stars, 36.2% (n=46) with a rating between 4.5 and 4.9 stars, and 7.9% (n=10) with a rating of a full 5 stars. A total of 18.9% (n=24) of the apps that we included had not been rated at the time of analysis.

### Archetypes of Motivational Design in Behavior Change Support Apps to Combat Procrastination

#### Overview

**Figure 3:**
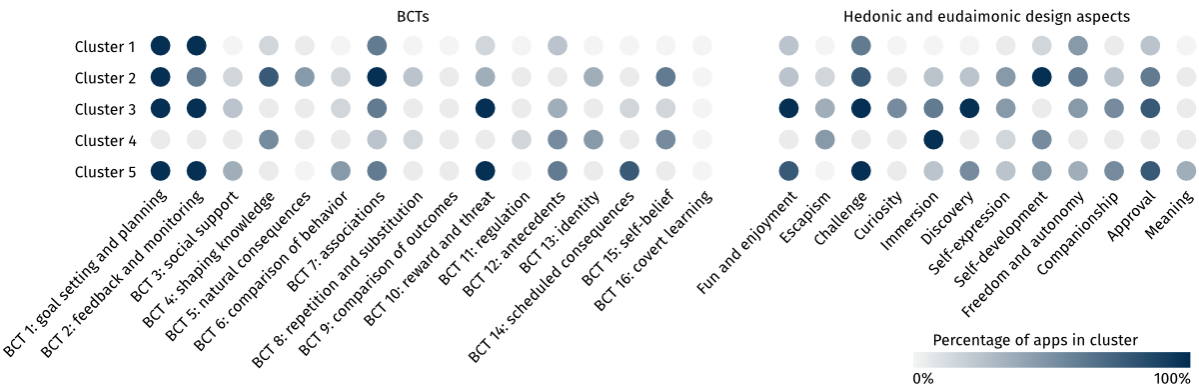
Graphical overview of the different clusters and the percentage of apps in each cluster that target specific behavior change techniques (BCTs; through functional design) and hedonic and eudaimonic design aspects.

Through our cluster analysis, we identified 5 distinct clusters of motivational behavior change apps to combat procrastination, which subsume a different number of apps. Specifically, cluster 1 was the largest cluster, with 51.2% (n=65) of the 127 apps, followed by cluster 2 and cluster 5, with 15.7% (n=20) of the apps each. Cluster 3 contained 10.2% (n=13) of the apps, and the smallest cluster, cluster 4, comprised 7.1% (n=9) of the apps. The apps in the clusters were rated fairly equally (mean 4.33, SD 0.55 stars for cluster 1; mean 4.30, SD 0.45 stars for cluster 2; mean 4.44, SD 0.31 stars for cluster 3; mean 4.42, SD 0.34 stars for cluster 4; and mean 4.55, SD 0.33 stars for cluster 5), with the apps in clusters 1 and 2 having the least positive ratings and the apps in cluster 5 having the most positive ratings. The clusters differed considerably in the BCTs and the hedonic and eudaimonic design aspects implemented in the contained apps (Figure 3). From the profiles of the clusters in terms of their motivational design, we can derive a motivational design *archetype* (ie, an abstract representation of a cluster), which helps understand how the clusters differ from each other. In the following sections, we will explain the 5 motivational design archetypes represented by the 5 clusters we found.

#### Archetype 1: Structured Progress Monitor

**Figure 4:**
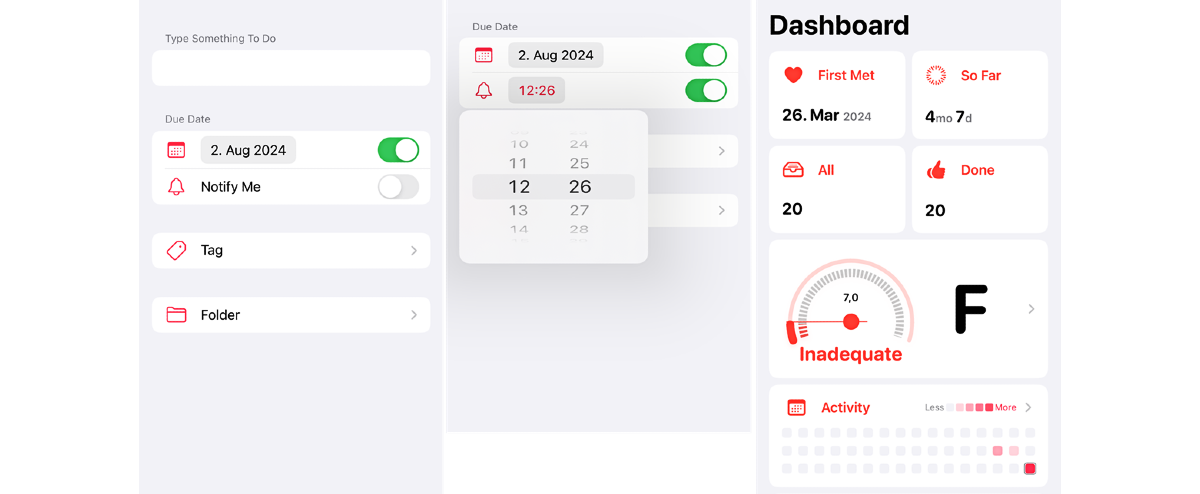
Noverdue as an exemplary app of the structured progress monitor archetype, with (A) the customizable creation of tasks, (B) the reminder function, and (C) the dashboard visualizing personal progress.

Archetype 1 represents an app used predominantly for managing personal tasks, goals, or habits. To establish goals and develop a plan of action (BCT 1), users can set tasks with a high degree of efficiency and ease. Furthermore, they can stipulate precise time frames for completion, the frequency of repetition, and the time of day at which the task is to be carried out. As a means of prompting users to engage with the app (BCT 7), notifications are sent to remind them to follow their task lists. The provision of detailed statistics and graphics for self-monitoring (BCT 2) represents a further key feature of the *structured progress monitor*. The visualization of one’s development, presented in the form of progress bars, grades, badges, points, or streaks, illustrates past achievements and the individual capacity to complete tasks successfully. These mechanisms elicit a sense of challenge in the user, prompting them to sustain the desired behavior and, consequently, adhere to the established goals over time. They represent the primary hedonic design aspect of the *structured progress monitor*, distinguished by a transparent and uncluttered design devoid of any significant hedonic or eudaimonic elements beyond the element of challenge. Consequently, it can be characterized as a primarily functional assistance system. An example of the *structured progress monitor* is the Noverdue app [79]. Figure 4 [79] illustrates how individuals can create tasks with notifications and due dates (Figure 4A [79]); personalize the reminder function (Figure 4B [79]); and self-monitor their progress on a dashboard that provides a rating, thereby challenging the users to improve their performance (Figure 4C [79]).

#### Archetype 2: Self-Improvement Guide

**Figure 5:**
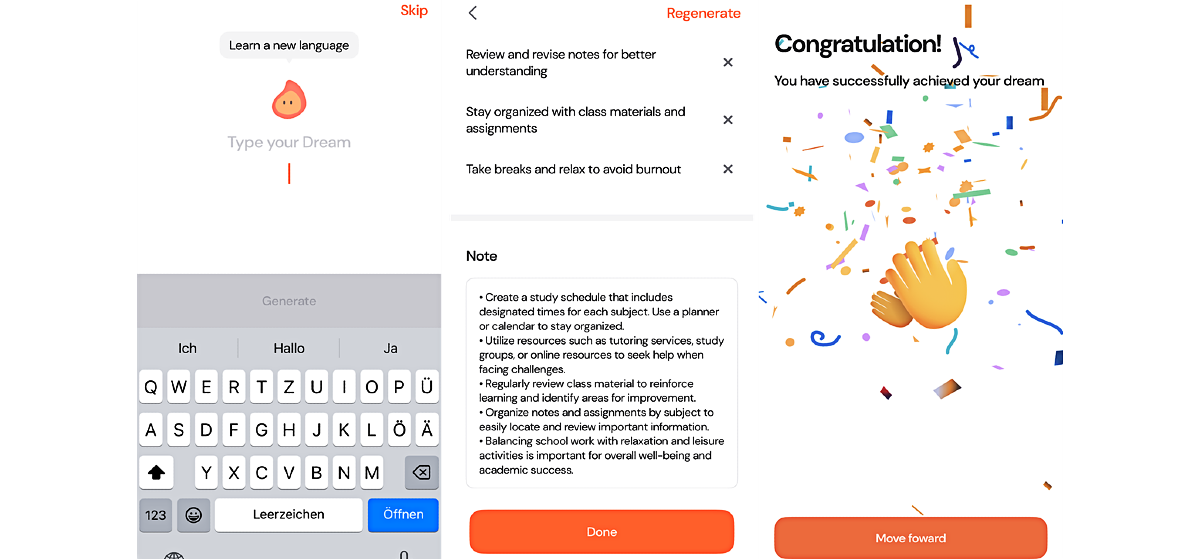
Dreamfora as an exemplary app of the self-improvement guide archetype, with (A) opportunities to define long-term goals and dreams, (B) the artificial intelligence assistance that breaks them down into tangible tasks and explains their relevance, and (C) ongoing approval for successful actions.

Archetype 2 provides the means to restructure detrimental behaviors and introduce novel habits (BCT 1) within one’s personal life. An artificial intelligence (AI)–powered chatbot offers tailored assistance and guidance (BCT 4) on overcoming obstacles and navigating questions regarding habit implementation while emphasizing the positive consequences of behavior change (BCT 5), for example, for personal well-being. This key mechanic is complemented by functionalities to track (BCT 2) and repeat (BCT 8) new habits and receive personalized reminders (BCT 7). In-app journaling encourages introspective reflection on one’s behavior, facilitating the development of self-belief (BCT 15). Consequently, the *self-improvement guide* assists users in articulating their desired personal development and provides guidance on how to achieve these life goals through the use of a digital companion, which offers continuous approval for positive self-development. The elicited feeling of self-development, in conjunction with challenge-oriented elements such as streaks, serves as the primary hedonic and eudaimonic mechanism that facilitates users’ maintenance of new habits. An illustrative example of the *self-improvement guide* is Dreamfora [46]. Figure 5 [46] illustrates how users can establish new, overarching objectives and dreams that they aspire to attain through self-development (Figure 5A [46]). The figure also depicts how the AI chatbot dispenses knowledge and assistance regarding forming new habits (Figure 5B [46]), thereby facilitating the achievement of the aforementioned life goals. Furthermore, it demonstrates how users receive ongoing approval for their successful actions (Figure 5C [46]).

#### Archetype 3: Productivity Adventure

**Figure 6:**
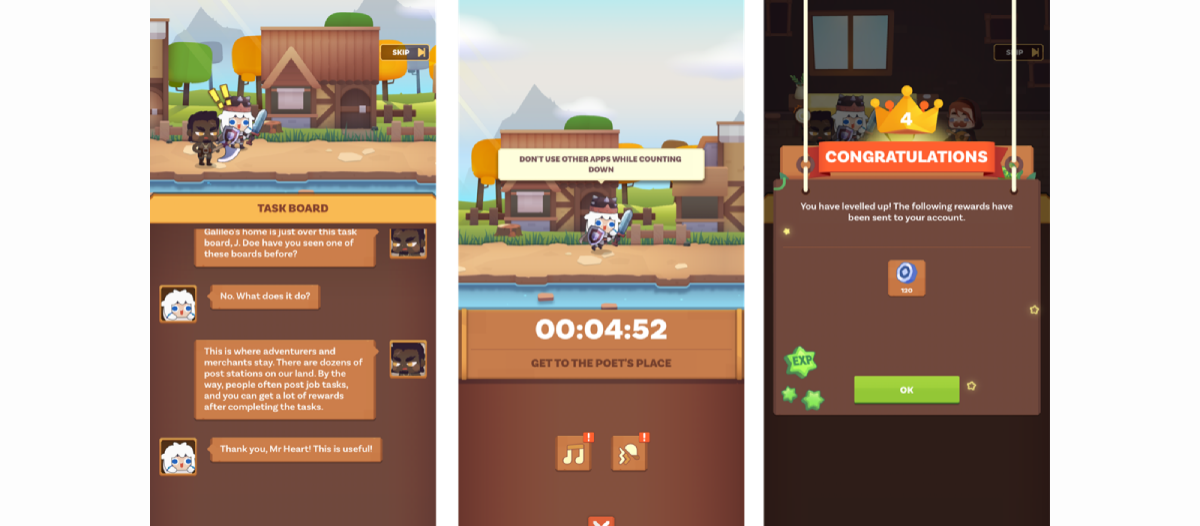
Focus Quest as an exemplary app of the productivity adventure archetype, with (A) a fictional storyline that entails the user’s character and other digital characters, (B) focus and task challenges, and (C) digital rewards and levels that support progress in the story.

Archetype 3 is distinguished by its comprehensive hedonic and eudaimonic design. Playfully designed storylines, mini games, unlockable levels, and customizable characters elicit feelings of enjoyment, challenge, curiosity, discovery, and self-expression among app users. By transforming the mundane and occasionally onerous task completion into a digital experience, the seriousness associated with task fulfillment is diminished. Digital characters that accompany the individual in the digital journal facilitate this process by offering a sense of companionship and providing approval. As a foundation for digital progression, the productivity adventure is predicated on 5 principal BCTs: the user defines their to-dos (BCT 1) and monitors the completion of these tasks in a step-by-step manner (BCT 2), aided by a timer that schedules focus sessions (BCT 12) and notifies upon successful completion (BCT 7). Should the user maintain their focus for the requisite period, the app will instantaneously reward them with, for example, the ability to access a new level (BCT 10). An example of this archetype is the Focus Quest app [80]. Figure 6 [80] illustrates the integration of goals and task fulfillment into a fictional storyline in interaction with a digital character (Figure 6A [80]), which challenges the user to maintain focus for a designated period (Figure 6B [80]) to obtain digital rewards and progress in the story (Figure 6C [80]).

#### Archetype 4: Emotional Wellness Coach

**Figure 7:**
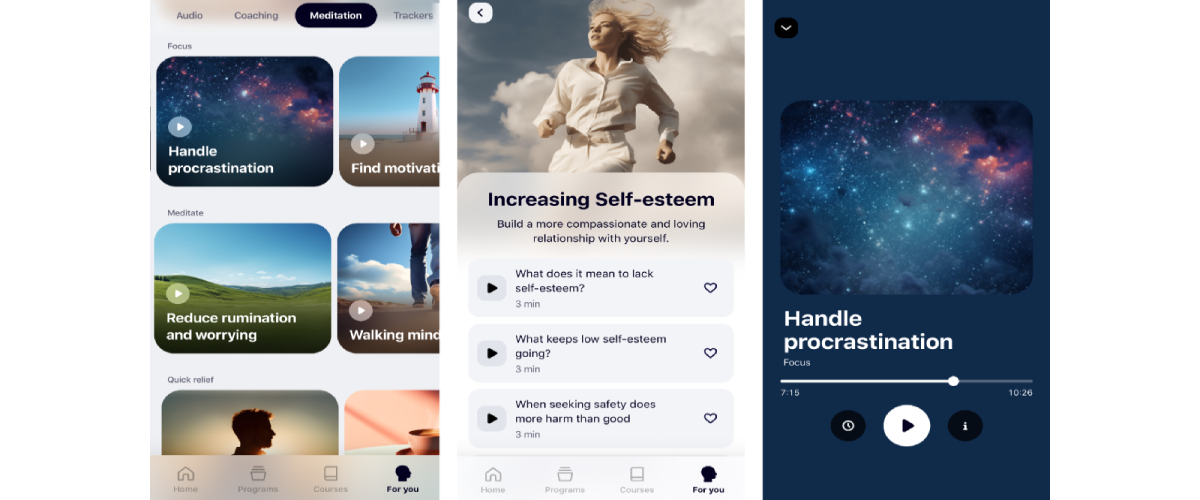
Greatness as an exemplary app of the emotional wellness coach archetype, with (A) a variety of meditation programs to choose from, (B) coaching sessions on motivation and emotional well-being, and (C) immersive meditation exercises.

Archetype 4 represents an app that furnishes users with a variety of content, including quotes, images, videos, and audio material, with the objective of enhancing motivation and emotional regulation, particularly in the context of perceived pressure. Positive affirmations, relaxing audio sessions, and motivational speeches by role models reinforce the user’s self-belief (BCT 15) and cultivate a positive outlook on one’s future identity (BCT 13). In terms of hedonic and eudaimonic design, the *emotional wellness coach* contributes to the attainment of a positive state of mind in which the user can escape the stressful reality and immerse themselves in meditative sessions, thereby preparing themselves mentally for pursuing their goals. This ultimately facilitates the process of intense cognitive self-development. An app that exemplifies the *emotional wellness coach* is Greatness [81], depicted in Figure 7 [81]. Individuals may select from an array of productivity- and well-being–oriented meditation programs (Figure 7A [81]). These programs comprise distinct coaching sessions to foster motivation and promote emotional well-being (Figure 7B [81]). Moreover, the app provides guidance and facilitates mental preparation and rehearsal through immersive meditation exercises (Figure 7C [81]).

#### Archetype 5: Social Focus Companion

**Figure 8:**
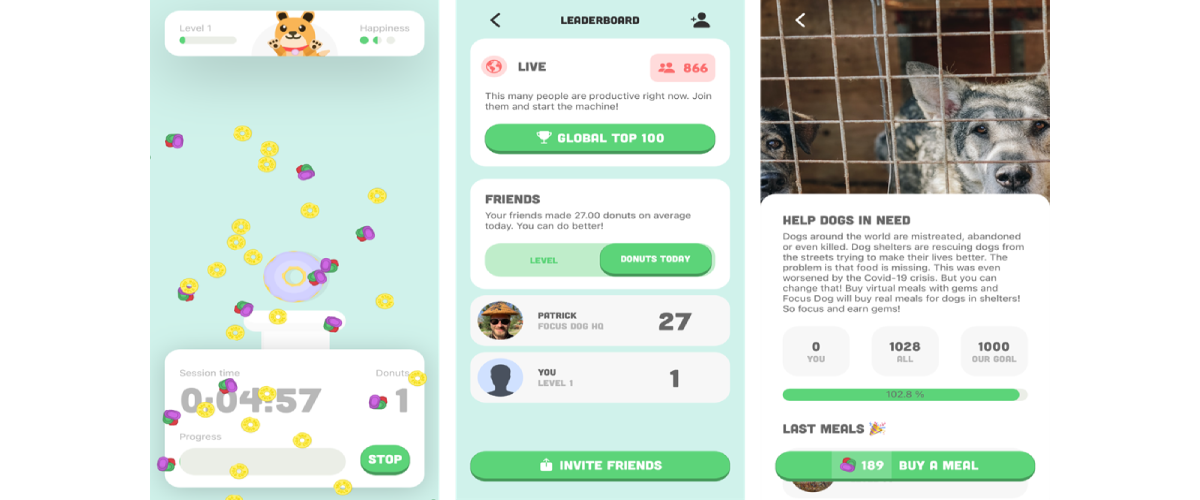
Focus Dog as an exemplary app of the social focus companion archetype, with (A) the fictional dog gaining donuts through focus time, (B) social comparison on a ranking list, and (C) the possibility to donate in-app coins to purchase meals for canines in need.

The primary operational mechanism of archetype 5 is a focus timer that inhibits the use of alternative apps and external distractions, thereby facilitating the control of environmental stimuli (BCT 12). The user establishes their to-dos along with an intended time interval (BCT 1). Upon the expiration of the designated time and the maintenance of focus, the user is granted the aforementioned scheduled rewards. Conversely, upon the occurrence of a distraction, the user is subjected to a digital penalty (BCTs 10 and 14). The primary hedonic design element is an in-app character situated in a playful and enjoyable environment. This character is developed and behaves in accordance with the user’s focus sessions. That is, it either progresses and flourishes through the digital rewards or suffers from the digital penalties. This dynamic challenges the user to complete focus sessions for the benefit of their digital companion. In this regard, the digital entity also offers feedback (BCT 2) indicating approval for successful work. Furthermore, in contrast to the other archetypes, the *social focus companion* allows for interaction with other users, facilitating social support in focusing together (BCT 3) and comparison with the behavior of others (BCT 6), fostering a feeling of companionship*.* While unlocking novel rewards also elicits a feeling of discovery, a distinctive eudaimonic feature of the *social focus companion* is the altruistic benefit of one’s behavior, evoking a sense of meaning. By adhering to the designated focus time, one not only rewards the character within the app but also achieves a positive impact outside the app. The exemplary app of the *social focus companion*, named *Focus Dog: The Productivity Timer* [82] (Figure 8 [82]), features a fictional dog that requires nourishment, which can be obtained by unlocking donuts through the use of coins (Figure 8A [82]). Individuals are rewarded with coins for maintaining focus, and users can compare their performance on a ranking list (Figure 8B [82]). Furthermore, in addition to benefiting the fictional dog, the coins can be used to purchase meals for canines in need (Figure 8C [82]).

## Discussion

### Principal Findings

#### Overview

Our study sheds a novel light on how practice has approached the design and implementation of motivational BCSSs for the unguided self-treatment of procrastination as a low-threshold alternative to professional medical treatment programs for those affected. Generally, while research on motivational BCSSs for procrastination is still nascent [17], we can observe that practice has already produced a plethora of behavior change support apps with various behavior change support techniques and hedonic or eudaimonic design aspects implemented to both provide functional support and elicit positive experiences in managing procrastination. In response to our research question, we identified 5 archetypes of motivational design in prevalent apps: the *structured progress monitor*, the *self-improvement guide*, the *productivity adventure*, the *emotional wellness coach*, and the *social focus companion*. In reflecting on our results, we can discuss 3 main observations that entail important implications for future research and practice in the field.

#### Observation 1: The Relationship Between Motivational Design Archetypes and Psychological Determinants of Procrastination

Upon juxtaposing the identified archetypes of motivational design with procrastination theory, it becomes evident that the designs appear to target different determinants of procrastination, specifically, outcome expectancies, outcome value, delay, and impulsiveness [2,12].

In particular, the *structured progress monitor* and the *productivity adventure* both appear to target primarily outcome value, that is, the desirability of a task or activity for an individual [2,12], and delay, which is a temporal indicator of the proximity of the outcome value’s realization [2,12]. In this regard, the *structured progress monitor* emphasizes task organization, self-monitoring, and the illustration of progress and self-development. From a theoretical perspective, the organization and planning of tasks (as a form of goal setting [83]) and adherence to a self-set schedule as a self-regulatory technique [2] affect procrastination through 2 mechanisms: temporal discounting and diminishing returns [12]. Specifically, this implies that the successful completion of each individual task that arises from a divided overall objective provides a similar level of satisfaction concerning the need for competence [84] to that for the entire project. Consequently, the overall outcome value accumulates as a determining factor in procrastination [12]. Simultaneously, individual tasks are more proximal than a distant deadline for an overall project, which reduces delay and, thereby, enhances the power of the accumulated outcome value [12]. The *productivity adventure*, similarly to the *structured progress monitor*, appears to seek to enhance the perceived outcome value of a task or action, albeit through a markedly different approach. By advancing in a narrative, obtaining digital rewards for completing tasks and focus sessions, and stimulating individuals’ curiosity and desire for exploration and discovery with unexpected events and unlockable paths and content, the archetype—in addition to merely subdividing a goal and illustrating progress—reframes task values by offering rewards and digital progress, thereby reducing task aversiveness [2]. Task unpleasantness and a lack of interest or boredom have been identified as reliable determinants of procrastination [10], reducing outcome value. Conversely, the satisfaction of autonomy needs through opportunities for exploration and discovery of a story or narrative [85] has been shown to counteract procrastination [86,87]. In addition, from a behavioral perspective, immediate (digital) rewards provide timely reinforcement of behavior [88-90], thereby reducing the delay of outcome value, particularly for individuals who are prone to immediate gratification [90].

In contrast, the *self-improvement guide* and the *emotional wellness coach* primarily aim to target outcome expectancies, that is, the conviction that a task can be completed successfully [2,12]. In this context, the *self-improvement guide* emphasizes the education of individuals regarding the consequences of procrastination and the means to counteract it. It provides support and approval for self-reflection, personal development, and the establishment of self-belief, thereby enabling those affected by procrastination to realize new habits (ie, behavioral routines vital in inhibiting procrastination [2,10]). From a theoretical perspective, research has emphasized that irrational beliefs, unrealistic expectations, and low self-esteem present critical risk factors for procrastination and a phenomenon called self-handicapping [10], which is defined as engagement in unrelated activities or tasks that are likely to fail [91]. The resolution of such cognitive barriers and the reinforcement of self-efficacy represent a crucial element in forming outcome expectancies [92]. In turn, this fosters a reduction in procrastination behaviors [12]. In a similar albeit different manner, the *emotional wellness coach* uses a cognitive approach to procrastination, eschewing the behavioral elements present in the other archetypes. This approach is centered on enhancing self-belief and identity through immersive formats and meditation while mitigating the pressure experienced by individuals. In this regard, procrastination theory demonstrates that pressure and stress resulting from perfectionism and fear of failure promote procrastination as a separate factor from self-esteem and self-efficacy [2,15]. The cognitive restructuring of perfectionist beliefs and mental contrasting sessions that highlight the steps necessary to achieve the goal rather than remaining in a passive mental state have been demonstrated to be effective therapeutic approaches to this problem [10]. These approaches are implemented in the emotional wellness coach as digital coaches or role models.

Finally, the *social focus companion* is primarily concerned with reducing distractibility and enhancing stimulus control. That is to say, it is focused on volitional rather than motivational factors [1]. Both are essential for strengthening self-control [2,92] and reducing impulsiveness, which is defined as a preference for short-term outcomes over future rewards [90] and presents a key determinant of procrastination [12]. To this end, the primary functionalities of the *social focus companion* are focus timers that mitigate distractions coupled with scheduled digital incentives and penalties for successful or unsuccessful focus sessions, respectively. These sessions are enhanced with playful environments, digital companions or assets, and linking of in-app achievements to meaningful additional outcomes. Moreover, the social comparison and support mechanisms inherent to the *social focus companion* reinforce the normative influence to focus on the task, which in turn reduces impulsiveness even further [14]. In addition, through a process known as *fusing*, immediate social gratification increases the perceived value of the outcome [2,10].

In summary, it can be observed that the *self-improvement guide* and the *emotional wellness coach* primarily target outcome expectancies as determinants of procrastination, whether through the enhancement of self-efficacy and self-belief or the reduction of perfectionism and pressure. The *structured progress monitor* and the *productivity adventure*, in turn, primarily seek to enhance outcome value by subdividing goals or reducing task aversiveness and decreasing delay through temporal discounting and immediate reinforcement. Finally, the *social focus companion* is concerned with reducing impulsiveness through stimulus control and normative influence. Therefore, from a procrastination theory perspective, the 5 archetypes address all the primary factors that contribute to procrastination [2,12].

#### Observation 2: BCTs in Motivational BCSSs for Procrastination Compared to Traditional Treatment Strategies

Research has suggested that BCSSs may provide an efficacious and low-threshold alternative to traditional therapeutic treatments for procrastination [11,14,17]. In this regard, it has been demonstrated that studies on BCSSs for procrastination rely heavily on behavioral treatment strategies, including self-supervision, planning and prioritization, time management, and goal setting [17]. However, cognitive approaches appear to be underrepresented and are predominantly used as an extension of cognitive behavioral therapy with professional guidance [17], also referred to as internet-based cognitive behavioral therapy [39,93,94]. Consequently, they are not yet widely regarded as a stand-alone self-treatment option. Our analysis of practical approaches to BCSSs to combat procrastination similarly demonstrates an emphasis on behavioral strategies, most importantly goal setting and planning (BCT 1), feedback and monitoring (BCT 2), and associations and cues (BCT 7). In this context, it is noteworthy that several archetypes, particularly the *productivity adventure* and the *social focus companion* but also the *emotional wellness coach*, incorporate functionalities to alter the natural environment and avoid distractions, targeting antecedents (BCT 12), whereas research has thus far primarily overlooked the potential of stimulus control, or volitional influences, in self-treatment BCSSs [17].

Furthermore, it can be observed that both the *self-improvement guide* and the *emotional wellness coach* use cognitive strategies, specifically knowledge shaping (BCT 4), identity building (BCT 13), and self-belief strengthening (BCT 15), through the use of predefined information and meditation sessions conducted by coaches or through AI companions that generate personalized recommendations based on the individual’s goals, challenges, and reflections. Although intelligent conversational agents can serve as a valuable alternative for individual coaching in other health care areas [95-97], their potential in research on BCSSs for procrastination remains unexplored [17,98]. Our analysis demonstrates that practice has already taken the initiative to use such approaches, which can potentially enable the tailoring of cognitive strategies to individuals and their specific problem profiles [1,56,57] for the purpose of self-treating procrastination. Scholars are encouraged to build on these implementations to further explore their potential for the personalized treatment of procrastination.

Nevertheless, when considering the therapeutic treatment of procrastination, it becomes evident that the prevailing self-treatment archetypes lack the incorporation of other cognitively oriented BCTs that have been demonstrated to exert a beneficial influence on procrastination. These include the comparison of outcomes through the reflection on the advantages and disadvantages of the behavior in question as well as the visualization of prospective outcomes (BCT 9) and emotional regulation (BCT 13) [99,100]. It remains unclear whether nonhuman artificial agents can entirely supplant therapeutic guidance, particularly regarding emotional regulation, which necessitates a personal bond [101] that is frequently impeded by cognitive and emotional barriers in the context of artificial systems [102,103]. Furthermore, medical research has demonstrated that group treatment and social strategies, including social support (BCT 3), comparison of behavior (BCT 6), and covert learning from others’ experiences (BCT 16), are more effective in treating procrastination than individual approaches [104]. In this regard, existing research efforts [17], as well as our identified practical archetypes, still demonstrate considerable potential for harnessing the power of social support. While the *social focus companion* notably uses joint focus sessions as a form of common goal setting and at least enables social comparison on ranking lists, such mechanics are primarily competitive [105] and oriented toward normative pressure that can evoke feelings of guilt or shame [106] rather than being socially supportive, as in the form of group counseling sessions and mutual exchange about the challenges and personal experiences with procrastination [107]. The combination of cognitive reflection and self-belief strategies with social support and covert learning may prove an effective approach to the self-treatment of procrastination. In particular, this approach may offer significant benefits for individuals facing challenges related to false expectations, irrational beliefs, low self-esteem, and other stable dispositions that require intensive cognitive restructuring [10] rather than behavioral support for task organization and stimulus control.

#### Observation 3: Hedonic and Eudaimonic Design of Motivational BCSSs for Procrastination

With regard to hedonic and eudaimonic design aspects of motivational design archetypes, it is evident that dominant designs are either centered on digital environments that are designed for enjoyment, featuring fictional characters and stories that foster discovery and provide a sense of approval (the *productivity adventure* and the *social focus companion*), or on providing means for users to escape pressures, engage in meditation, and support their self-development (the *self-improvement guide* and the *emotional wellness coach*). It is noteworthy that practice has used a more diverse array of hedonic and eudaimonic design elements than research efforts in the field of motivational BCSSs for procrastination, which have primarily focused on challenge-oriented elements such as points [43,108,109] and badges [109,110], similarly to the *structured progress monitor* [17]. Furthermore, in comparison to most existing works on motivational BCSSs for other health dimensions beyond procrastination, such as physical activity [23,50] or therapy adherence [111], our identified practical archetypes extend beyond the use of challenge-oriented elements commonly observed in classic gamification [112]. They incorporate exploration, narrative design, and immersive elements, which have been recently proposed by research on motivational BCSSs to enhance the hedonic and eudaimonic experience effects beyond those typically associated with challenge-oriented approaches [113,114].

However, when considered together with the treatment strategies, cognitive approaches appear to be at odds with fictional environments and narratives in the identified archetypes. Thus far, motivational design archetypes seem to be focused on either using playful reframing to enhance hedonic and eudaimonic experiences during behavioral strategies or on fostering feelings of self-development and escapism from pressures during cognitive strategies. The latter naturally focus on real-life problems and the individual rather than a fictional story. In this regard, research on motivational BCSSs in other areas indicates that narrative design can be effectively combined with self-reflection exercises, which may enhance the beneficial hedonic and eudaimonic experiences elicited by both approaches [115-117].

Furthermore, novel motivational BCSSs that extend beyond the smartphone technology examined in this study, such as virtual reality applications, may offer even greater potential to integrate immersive narratives with cognitive treatment approaches, a relaxing environment, and feelings of actual self-development [118,119]. While those developments are still in their infancy, both research and practice can benefit from exploring such novel technological advancements in designing effective motivational BCSSs for procrastination.

### Implications

The principal findings of our study have significant implications for further research and practice. From a research perspective, our findings identified 5 main archetypes of motivational design in BCSSs for procrastination. These archetypes constitute a novel perspective on design knowledge for motivational BCSSs that abstracts from single, potentially ineffective real-world systems [48] and describes industry best practices that have prevailed in the design of motivational BCSSs for procrastination. For researchers, these archetypes provide a unique opportunity to obtain a holistic picture of how BCTs and hedonic and eudaimonic design elements have predominantly been instantiated in apps for procrastination. Researchers may juxtapose these findings with their own research endeavors, thereby potentially identify mismatches between current research foci and industry best practices. Consequently, they may discover new research opportunities or necessities to reallocate resources toward investigating the archetypal design configurations as possibly promising combinations of BCTs and motivational design that extend beyond the predominant focus on behavioral approaches [17].

Moreover, our observations indicate that the different motivational design archetypes may be suitable for targeting different determinants of procrastination [12]. Specifically, we found that apps in the *self-improvement guide* or *emotional wellness coach* archetypes primarily target outcome expectancies, whereas the *structured progress monitor* and *productivity adventure* seek to enhance outcome value and the *social focus companion* focuses on reducing impulsiveness [12]. While the archetypes were identified based on apps in the US and German markets and, therefore, predominantly reflect Western design approaches to target procrastination, research indicates that the primary determinants of procrastination, such as outcome expectancies, task aversiveness, and impulsiveness or self-control, are applicable and relevant across cultural contexts that are different from the Western one, including those of countries such as China, India, Israel, and Turkey [120-123]. Consequently, our design archetypes constitute a valuable foundation for further research, not only within the Western cultural context but, more broadly, to explore the potential of BCSSs as a low-threshold self-treatment approach for the global phenomenon of procrastination [2,3] across cultures. For example, given that individuals differ in their procrastination profiles and which of the determinants pose the most significant problem for them [1,56,57], there is great potential to examine how these motivational BCSS archetypes can serve as the basis for targeted self-treatment strategies for different individuals. The existing research has already called for implementing more individualized treatment strategies for procrastination [1,44]. Concurrently, the extant motivational BCSS research has demonstrated that, in addition to the treatment approach, individuals also express preferences for various hedonic and eudaimonic design elements that have the potential to influence their experiential impact [124-126]. Our findings provide a foundation for further research into how the different motivational design archetypes, in terms of both their treatment focus (behavioral, cognitive, or a combination) and their hedonic and eudaimonic design approaches, affect individuals with varying procrastination problem profiles, dispositions, situational contexts, and motivational preferences. In this way, targeted recommendations can be developed for individuals affected by procrastination.

Furthermore, our 5 motivational design archetypes present a blueprint for the effective practical development of BCSSs for procrastination. By demonstrating how designers can use and combine hedonic and eudaimonic design elements to enhance the efficacy of behavioral and cognitive BCTs in digital applications [40,41] while concurrently addressing dropout rates [58] and how designers can build on our archetypes as established configurations of design elements [49] rather than experimenting with inefficient combinations of BCTs [48], our study paves the way for an even more diverse array of motivational BCSSs to combat procrastination. Potential avenues for exploration include the integration of AI-based conversational agent approaches to enhance cognitive treatment strategies [95-98] and the use of virtual reality technology to unify cognitive and behavioral treatment and playful and narrative designs [127] with the objective of enhancing both the functional and experiential aspects of motivational BCSSs for procrastination. From the perspective of health professionals and affected individuals, our findings provide a basis for selecting a motivational design archetype that best suits the patient’s or individual’s challenges, needs, and preferences. Once this has been done, a suitable, existing app can be chosen from the results of our systematic cluster analysis. Consequently, our study provides a foundation for potentially enhancing the treatment adherence of individuals seeking mobile apps to assist them in addressing procrastination (as opposed to, eg, experiencing frustration due to the absence of effects from attempting to use an emotional wellness coach when the primary issue lies in stimulus control and distractibility). Therefore, ultimately, our findings assist in counteracting the significant mental and physical health consequences associated with procrastination [5-9].

### Limitations and Future Research

It is essential to consider some critical limitations in interpreting the findings of this study, which have their roots in the methodological choices made.

First, we restricted our examination of motivational BCSSs against procrastination to smartphone-based apps. Despite this decision, our search and screening process demonstrates that we identified a multitude of motivational behavior change apps for procrastination. However, we recognize that there are other motivational BCSSs for the self-treatment of procrastination that rely on alternative technology, such as web applications [128], which were excluded from our cluster analysis. Furthermore, while the Apple App Store and the Google Play Store represent the 2 largest app stores for iOS- and Android-based mobile phones, there are other app stores for Android-based mobile phones, such as the Microsoft Store, that were not included in our search. Similarly, it is crucial to acknowledge that user settings, filters, and preferences in app store searches may impact the results obtained by entering identical search terms on disparate devices. Consequently, some users may not be able to access all the apps that we screened for use on their smartphones. In addition, it should be noted that our search and screening were limited to German or English search terms and apps because the United States is the most significant Western mobile app market in terms of app downloads and consumer spending on mobile devices [60] and Germany represents the largest mobile app market in the European Union [61]. As a result, it is possible that we overlooked valuable apps that were designed for the Chinese market and only accessible and functional with Chinese-language proficiency.

Furthermore, while our attention was directed toward motivational BCSSs due to their hedonic and eudaimonic design characteristics that can augment positive experiences [40,41] and, in turn, potentially increase self-treatment adherence, it is important to recognize that, within the realm of research, numerous BCSSs aimed at combating procrastination have demonstrated efficacy despite not relying on hedonic or eudaimonic design [17]. In this regard, our archetype analysis provides a foundation upon which the effects of different motivational design approaches can be investigated; however, it does not provide insights regarding their effectiveness itself. It is recommended that future research be conducted to build on this open avenue and explore the distinct effects of different motivational design archetypes compared to merely functional BCSSs for procrastination.

Ultimately, in recognizing that most studies in the domain of BCSSs for procrastination that rely on cognitive behavioral treatment strategies use BCSSs to augment existing therapeutic guidance [17], we restricted our cluster analysis to exclusively self-treatment motivational BCSSs, omitting the inclusion of therapeutic guidance. Although our objective was to concentrate on apps that have the potential to be low-threshold alternatives to therapeutic treatment [11,14], it is important to acknowledge that professional assistance may be essential and irreplaceable for individuals who are already experiencing serious mental and physical health consequences of procrastination. In such cases, it could even be irresponsible to provide them with a stand-alone solution that is not designed as a medical product. Consequently, an avenue for future research in the treatment of procrastination is the analysis of the design opportunities for hybrid apps that provide hedonic and eudaimonic experiences while simultaneously integrating professional therapeutic guidance.

### Conclusions

Although procrastination represents a significant self-regulatory failure with the potential to cause adverse mental and physical health consequences, the rate of adherence to professional therapies remains low, particularly in light of the considerable number of individuals affected. Specific forms of BCSSs, namely, motivational BCSSs that elicit positive experiences through hedonic and eudaimonic design elements, have been proposed as a promising self-treatment alternative for individuals struggling with procrastination. However, research on the design and effects of such systems is still in its infancy. Conversely, a multitude of motivational behavior change support apps designed to facilitate behavior change and overcome procrastination have already been developed and implemented in practice. To analyze these practical developments, a systematic review and cluster analysis of prevalent smartphone apps was conducted, resulting in the identification of 5 motivational design archetypes. Upon critical discussion, these archetypes appeared to target different psychological determinants of procrastination and successfully use a variety of hedonic and eudaimonic design elements that extend beyond the current state of research. Nevertheless, further investigation is warranted to ascertain how hedonic and eudaimonic design can be more effectively integrated with cognitive BCTs and how social support mechanisms that have demonstrated efficacy in traditional procrastination treatment can be elicited through motivational BCSSs.

## Appendix

Multimedia Appendix 1 App list.

Multimedia Appendix 2 Cluster analysis results.

## Abbreviations

AI: artificial intelligence
BCSS: behavior change support system
BCT: behavior change technique
BCTT: Behavior Change Technique Taxonomy
IS: information system
PSD: persuasive system design
